# The Major Histocompatibility Complex of Old World Camels—A Synopsis

**DOI:** 10.3390/cells8101200

**Published:** 2019-10-05

**Authors:** Martin Plasil, Sofia Wijkmark, Jean Pierre Elbers, Jan Oppelt, Pamela Anna Burger, Petr Horin

**Affiliations:** 1 Department of Animal Genetics, Veterinary and Pharmaceutical University, Palackeho trida 1, 612 42 Brno, Czech Republic; plasilma@vfu.cz (M.P.); wijkmark.sofia@gmail.com (S.W.); 2 Ceitec VFU, RG Animal Immunogenomics, Palackeho trida 1, 612 42 Brno, Czech Republic; 3 Research Institute of Wildlife Ecology, Department of Integrative Biology and Evolution, Vetmeduni Vienna, Savoyenstraße 1, 1160 Wien, Austria; jeanpierre.elbers@vetmeduni.ac.at (J.P.E.); pamela.burger@vetmeduni.ac.at (P.A.B.); 4 Ceitec MU, Masaryk University, Kamenice 753/5, 625 00 Brno, Czech Republic; jan.oppelt@mail.muni.cz; 5 Faculty of Science, National Centre for Biomolecular Research, Masaryk University, Kamenice 753/5, 625 00 Brno, Czech Republic

**Keywords:** MHC, major histocompatibility complex, Old World camels, camels, dromedary, Bactrian camel, SNP

## Abstract

This study brings new information on major histocompatibility complex (MHC) class III sub-region genes in Old World camels and integrates current knowledge of the MHC region into a comprehensive overview for Old World camels. Out of the MHC class III genes characterized, *TNFA* and the LY6 gene family showed high levels of conservation, characteristic for MHC class III loci in general. For comparison, an MHC class II gene *TAP1*, not coding for antigen presenting molecules but functionally related to MHC antigen presenting functions was studied. *TAP1* had many SNPs, even higher than the MHC class I and II genes encoding antigen presenting molecules. Based on this knowledge and using new camel genomic resources, we constructed an improved genomic map of the entire MHC region of Old World camels. The MHC class III sub-region shows a standard organization similar to that of pig or cattle. The overall genomic structure of the camel MHC is more similar to pig MHC than to cattle MHC. This conclusion is supported by differences in the organization of the MHC class II sub-region, absence of functional DY genes, different organization of MIC genes in the MHC class I sub-region, and generally closer evolutionary relationships of camel and porcine MHC gene sequences analyzed so far.

## 1. Introduction

The major histocompatibility complex (MHC) is a genomic region of critical importance for vertebrate immune functions. Class I and II MHC molecules are responsible for the presentation of peptides to T cells of intracellular and extracellular origin, respectively [1]. Different MHC molecules encoded by different alleles of multiple MHC genes can bind and present different groups of structurally similar antigenic oligopeptides. Compared to highly specific T- and B-cell receptors, MHC molecules are less specific. This phenomenon allows MHC molecules to cope with the immense diversity of protein antigens based on inherited genetic variation. Different antigen presenting molecules are encoded by different allelic variants of MHC class I and class II genes. The MHC region is characterized by many loci with immune as well as with non-immune functions. Of these, multiple loci code for antigen presenting molecules that possess many alleles and have higher heterozygosity typically than predicted by neutrality [2]. As a result, the MHC is one of the most complex and most polymorphic regions of the mammalian genome [3]. Both parasite-mediated and sexual selection shape functional MHC polymorphism in mammalian populations [4]. However, different taxonomic families, and even different species within a family, have evolved various strategies allowing them to successfully cope with the selection pressure of extremely variable pathogens and with other environmental challenges [5,6,7,8]. Therefore, further studies of mammalian species and families are needed for obtaining a comprehensive and unbiased picture of MHC function and diversity [9].

The first camelids appeared in North America, 45–40 Mya [10]. While the ancestors of New World camelids migrated to South America, the ancestors of Old World camelids migrated into Asia (around 6–7 Mya) and continued to spread further west. It is now generally accepted that the divergence between one-humped dromedary (*Camelus dromedarius*) and two-humped Bactrian (*Camelus bactrianus*) camel occurred sometime between 4 and 7 Mya [11,12]. Due to the advancements in camel evolutionary studies, it is well established that extant Old World camelids are composed of three species – *Camelus dromedarius*, *Camelus bactrianus* and *Camelus ferus* [11,12,13,14]. The other recent part of the *Camelidae* family is represented by New World camelids, which include alpaca (*Vicugna pacos*), vicugna (*Vicugna vicugna*), llama (*Lama glama*) and guanaco (*Lama guanicoe*).

Camels are an economically and socially important species for a large part of the world. Since ancient times, they have been used as animals of burden, for milk and meat production, as well as for riding and racing. They represent a sustainable livestock species with several specific features [15]. In addition to their adaptation to the arid and dusty environment of deserts, such as specialized water and fat metabolism, stress response to high temperatures, and adaptation to intense ultraviolet radiation, they are more resistant to infectious diseases when compared to other species living in the same geographical area, and they also produce milk with a high content of antimicrobial agents. [16,17,18].

While genome and transcriptome analyses reveal genes underlying the adaptations of camels to the harsh environment [11,19], another specific feature of camels, their immunogenome, has been only poorly studied so far [11,15]. Camelids are the only mammals producing heavy-chain homodimeric antibodies without a light chain and with the antigen-binding fragment reduced to a single heavy chain variable domain [20]. Similarly, camel T-cell receptors and their genes are quite unique in terms of their structure and function, especially the TR γ/δ loci. In dromedary, (*Camelus dromedarius*), γ/δ rearranged genes, somatic hypermutation increases repertoire diversity, very much similar to the process of affinity maturation of immunoglobulin genes [21].

In this context, it is surprising that only little attention has been paid to MHC studies not only in Old World camels but in all camelids. The first information on the existence of MHC in dromedary and alpaca genomes was provided by Antczak [22], using cytogenetic techniques. In Old and New World camelids, the MHC is located on the long arm of chromosome 20 [23,24], and the first characterization of dromedary and Bactrian MHC class II and class I genes was provided by Plasil et al. [24,25]. The most common structure of the mammalian MHC region, represented by the order MHC class II–MHC class III–MHC class I genes, was identified in camels as well [24]. The organization of the MHC genes within these regions also follows the general pattern observed in other mammalian species [25]. A low level of polymorphism of the class II genes *DRA*, *DRB* and *DQA* genes was observed in all three Old World camel species, as well as for class I genes [24,25]. Surprisingly, MHC class I related *MR1* and *MICA* loci were found to be more polymorphic than a classical MHC class I locus, *B-67* [25].

Considering the gaps in the so far studied general organization of the camel MHC, and based on a new dromedary genome assembly [26], the aim of this work was to characterize selected MHC class III genes, and to integrate the new and previously published knowledge into an improved overview of the MHC region with a special focus on *Camelus dromedarius*. For this purpose, MHC-encoded molecules with different functions were selected. *TNFA* was chosen as an example of a typical, well conserved MHC class III gene, while *the LY6G6* genes were selected as a family of genes with a common origin and located in close physical proximity. *TAP1* classified as an MHC class II gene, was selected to determine variation of gene encoding non-antigen presenting molecules, and to compare it with variation observed for the studied MHC class II [24] and MHC class III genes.

## 2. Materials and Methods

### 2.1. Analysis of Selected Non-Antigen Presenting MHC Genes

The camel DNA samples used in this study come from collections at the Research Institute of Wildlife Ecology, Vetmeduni Vienna and UPVS Brno. Bactrian camel samples were collected at three different locations in Mongolia and one sample was collected from a breeder in Austria, while dromedary samples were from Jordan, Saudi Arabia, UAE, Qatar, Sudan, Kenya, Kazakhstan and Nigeria. Numbers of analyzed camels of both species are presented in Table 1. All samples were collected commensally during veterinary procedures for previous research projects (GACR 523/09/1972; PI: P. Horin; FWF P1084-B17 and P24706-B25; PI: P. Burger).

An MHC class II gene *TAP1*, and MHC class III *TNFA,* and the *LY6G6* gene family were selected for this study. All these genes were already annotated. We amplified and sequenced them as described previously for MHC class I genes [25]. Briefly, individual gene sequences were extracted from sequence resources at NCBI, as seen in Tables S1–S6. Primers were designed using either the Primer3 Webtool or NCBI PrimerBLAST in Table 2. PCR was performed according to standard protocol using either KAPA 2G Robust HS or KAPA LongRange HS (KapaBiosystems, Wilmington, DE, USA). Sequencing was performed on Illumina MiSeq platform (San Diego, CA, USA) using 500 cycles PE chemistry. Data analysis was performed as previously described in Plasil et al. [25].

All SNP positions are numbered according to their respective reference sequences; for all loci, their accession numbers can be found in supplementary files.

### 2.2. New Annotation of the MHC Region and Phylogenetic Analyses

We used the newly available resource of the *C. dromedarius* genome assembly CamDro3 [26], which improves upon work published by Elbers et al. [27], for refining our previous characterization of the MHC region to produce a detailed map of all three MHC class regions [24,25]. The CamDro3 assembly is a result of upgrading the CamDro2 assembly, similarly to Elbers et al. [27], where CamDro1 assembly was upgraded to CamDro2 [28]. The CamDro2 assembly was re-scaffolded using the original Dovetail Chicago and Hi-C reads with the HiRise pipeline [29] in an attempt to fix local misassemblies. We then filled in gaps using our PacBio long-reads (SRA accession: SRP050586) [27] with PBJelly v. 15.8.24 [30] twice instead of one time, which was done for CamDro2. Instead of polishing the assembly with Pilon [31], we used a standard variant calling workflow, which increased the RNA-Seq mapping rates relative to the Pilon-polished assembly. Briefly, we first mapped, trimmed and error-corrected Illumina short-insert sequences (SRA accession: SRR2002493) [28], using BBMap v. 38.12 (https://sourceforge.net/projects/bbmap/) with the vslow and usejni settings to the PBJelly assembly. We then sorted and indexed the resulting BAM file with Sambamba v. 0.6.7 [32], and called variants with CallVariants v. 38.12 (https://sourceforge.net/projects/bbmap/). We finally used BCFtools v. 1.2 (http://samtools.github.io/bcftools/) to generate a consensus sequence, for which we filled in gaps using ABYSS Sealer v. 2.1.0 [33], using default settings except for a bloom filter size of 40 GB, and multiple *K* values from 90 to 20 in increments of 10.

For phylogenetic analyses of the MHC genes studied here, we included annotated sequences from other mammalian species available at NCBI, as seen in Tables S1–S6. Phylogenetic trees were constructed in MEGA7 using the maximum-likelihood (ML) approach with 1000 bootstraps and the best-fitting model was tested according to the Bayesian information criterion (BIC) [34]. I.e., the best evolutionary model for *TAP1*, *TNFA, LY6G6E* and *LY6G6F* followed the Tamura 3-parameter with gamma distribution (5 categories with parameters 0.5098, 0.1968, 0.5569 and 0.8816, respectively) [35]. The *LY6G6C* and *LY6G6D* trees were based on the Jukes-Cantor and Kimura 2-parameter [36,37], respectively.

We have constructed a schematic overview of the MHC regions of selected species to compare their general similarities as well as to summarize evolutionary relationships between individual genes. For this purpose, we used the above-mentioned dromedary assembly CamDro3 as well as assemblies of the following species: Cattle (ARS-UCD1.2 (GCF_002263795.1)), pig (Sscrofa11.1 (GCF_000003025.6)), dog (CanFam3.1 (GCF_000002285.3)), human (GRCh38.p12 (GCF_000001405.38)), and data from our previous studies [24,25,38].

We have also prepared a synteny plot to directly compare the MHC regions of camelids. We lifted over NCBI annotations (release 101) for the *V. pacos* assembly GCF_000164845.2 to the DNA Zoo *V. pacos* assembly (https://www.dnazoo.org/assemblies/Vicugna_pacos) with flo (https://github.com/wurmlab/flo). We then extracted the MHC region (sequences and annotations between genes *RXRB* and *TRIM27*) for the DNA Zoo *V. pacos* assembly and the MHC regions for *C. dromedarius* (CamDro3 assembly), *C. bactrianus* (CamBac2 assembly, GCF_000767855.1 scaffolded with CamDro3), and *C. ferus* genomes (CamFer2 assembly, GCF_000311805.1 scaffolded with CamDro3) [26]. Annotations for CamDro3, CamBac2, and CamFer2 were generated with MAKER v. 2.31.10 following an approach similar to Elbers et al. [27,39,40]. We generated a synteny plot of the MHC regions for *V. pacos* (DNA Zoo V. pacos), *C. bactrianus* (CamBac2), *C. dromedarius* (CamDro3), and *C. ferus* (CamFer2) with Synima [41].

## 3. Results

### 3.1. The MHC Class II Gene TAP 1

Compared to the available annotated coding sequences (CDSs) of Old World camels (XM_010994582.1:25-2265), one substitution was found in the CDS of the *TAP1* gene. Out of the total of 19 SNPs within the CDS, 12 were non-synonymous and 7 were shared between *Camelus bactrianus* and *Camelus dromedaries*, as seen in Table S7, Figure S1. We detected a discrepancy between the here developed *TAP1* CDS and the sequences available at NCBI, as seen in Table S1. While the alignment of the published dromedary and Bactrian camel sequences showed significant differences between sequence positions 520-585 of the CDS, we could not confirm them in our sequences as all analysed sequences bear close similarity to the sequence of *C. dromedaries*, as seen in Figure S2. A potential deletion between positions 265 and 306 bp was identified in the CDS of *C. ferus* obtained from NCBI, as seen in Table S1.

**Figure 1:**
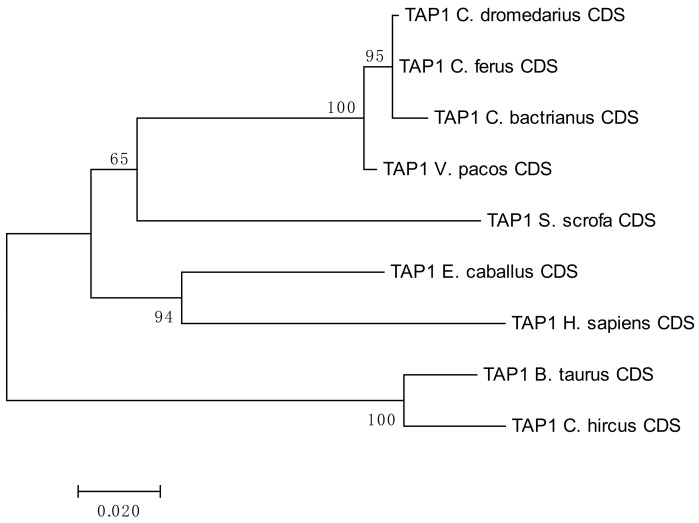
Phylogenetic tree of selected *TAP1* coding sequences (CDSs). The tree is based on the maximum-likelihood method using Tamura 3-parameter model with gamma distribution (five categories, parameter = 0.5098). Number of bootstraps = 1000.

A phylogenetic analysis of the camel *TAP1* sequences showed high level of conservation within *Camelidae*. Despite its lower bootstrap support, it suggested a relatively close evolutionary relationship with the domestic pig, *Sus scrofa*, as seen in Figure 1. Additional information regarding the sequence similarity of the analyzed *TAP1* CDSs is available in the Table S1.

### 3.2. MHC Class III Genes

#### 3.2.1. The TNFA Gene

**Figure 2:**
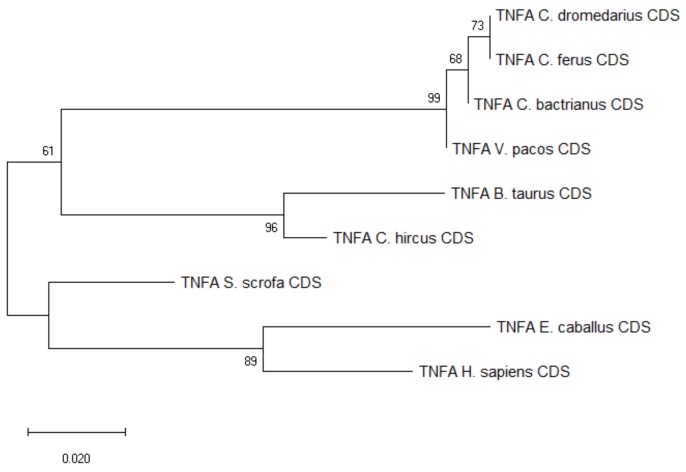
Phylogenetic tree of the *TNFA* CDSs from selected species. The maximum-likelihood method was used based on the Tamura 3-parameter model with gamma distribution (five categories, parameter = 0.1968). The number of bootstraps was 1000.

The three SNPs found within the *TNFA* CDS in exon 2 (1 non-synonymous) and exon 3 (1 synonymous, 1 non-synonymous) were shared between *C. bactrianus* and *C. dromedaries*, as seen in Figure S3. The haplotypes were conserved across the entire CDS, i.e., the three SNPs were either in homozygous or heterozygous constitution, and the frequency of heterozygote individuals was 0.48 and 0.27 in dromedaries and Bactrian camels, respectively. A comparison with other publicly available camelid sequences did not reveal any additional potential SNPs, as seen in Figure S4. The phylogenetic tree showed an overall high level of conservation within *Camelidae,* and closer evolutionary relationships with sequences from cattle and goat, as seen in Figure 2. Additional information regarding sequence similarity of the analyzed *TNFA* CDSs is available in the Table S2.

#### 3.2.2. The LY6G6 Gene Family

Four genes of this family, *LY6G6C, LY6G6D*, *LY6G6E*, and *LY6G6F* were analyzed and have been annotated in the three species of Old World camelids. While we discovered only one non-synonymous SNP within the *LY6G6C* CDS at position 218 (G/A) present in *C. dromedaries,* as seen in Figure S5, the sequences retrieved from NCBI showed another non-synonymous SNP at position 235 (C/A) as seen in Figure S6. However, this SNP was not confirmed by our dataset. The frequency of heterozygotes for the *LY6G6C* SNP 218 in *C. dromedarius* was 0.2. The CDS of the *LY6G6D* contained one synonymous SNP (G/T at position 405) observed only in *C. bactrianus,* as seen in Figure S7. The frequency of heterozygotes for this SNP was 0.2 in *C. bactrianus*. Comparison with other available sequences from NCBI did not reveal any additional SNPs in Old World camelids, as seen in Figure S8.

In the family *Camelidae*, the *LY6G6E* gene has been annotated as *sperm acrosome membrane-associated protein 4-like*. Figure S9 shows our analyses, as well as sequences retrieved from NCBI, that the *LY6G6E* CDSs were monomorphic in the subfamily *Camelini*. However, a comparison with the CDS of *Vicugna pacos* (*Lamini*) revealed two SNP positions (319 T/C and 358 G/A), where the latter was non-synonymous. Comparison with the CDS of *Bos taurus* showed that the CDSs compared were not of the same length.

One synonymous (A/G, at position 150) and one non-synonymous SNP (C/T, at position 659) were identified in the CDS of *LY6G6F*, both shared between *C. bactrianus* and *C. dromedaries*, as seen in Figure S10. The frequencies of heterozygotes in *C. bactrianus* and *C. dromedarius*, for both SNPs were 0.3 and 0.5, respectively. Individuals of both species were either heterozygous or homozygous in both SNPs, but never homozygous for one and heterozygous for the other. Comparison of the sequences available on NCBI confirmed both SNPs, as seen in Figure S11.

**Figure 3:**
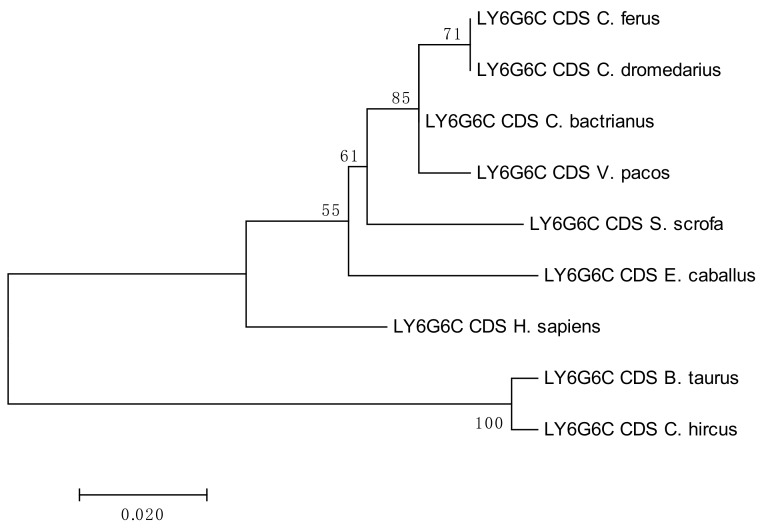
Phylogenetic tree of the *LY6G6C* CDSs from selected species. The maximum-likelihood method was used based on the Jukes-Cantor model. The number of bootstraps was 1000.

**Figure 4:**
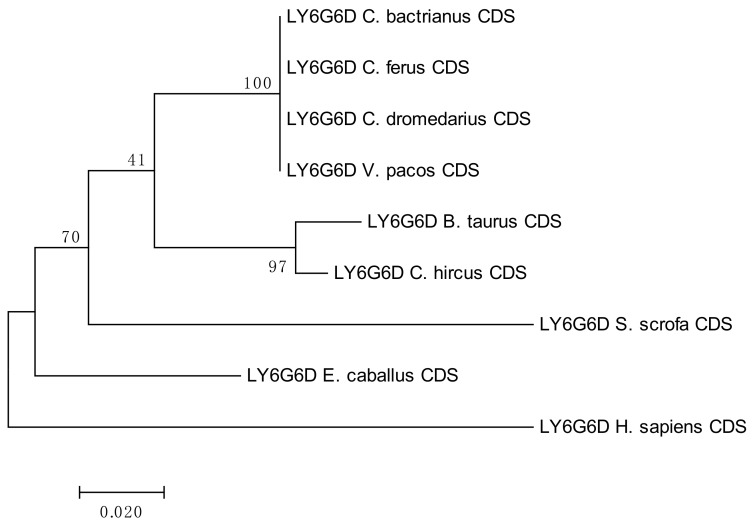
Phylogenetic tree of the *LY6G6D* CDSs from selected species. The maximum-likelihood method was used based on the Kimura 2-parameter model. The number of bootstraps was 1000.

**Figure 5:**
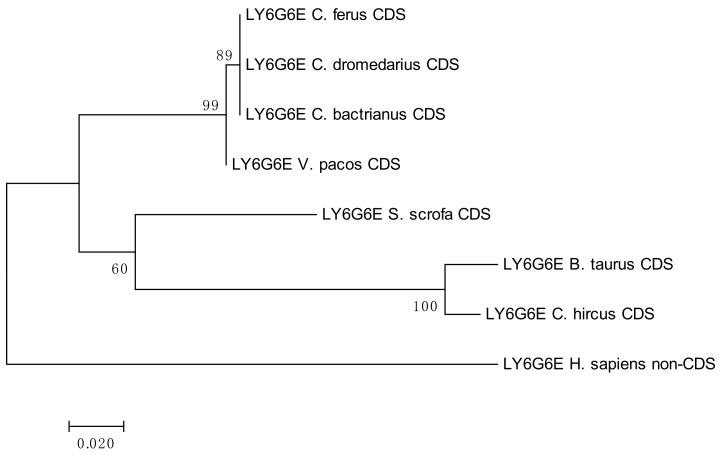
Phylogenetic tree of the *LY6G6E* CDSs from selected species. The maximum-likelihood method was used based on the Tamura 3-parameter model with gamma distribution (five categories, parameter = 0.5569). The number of bootstraps was 1000.

**Figure 6:**
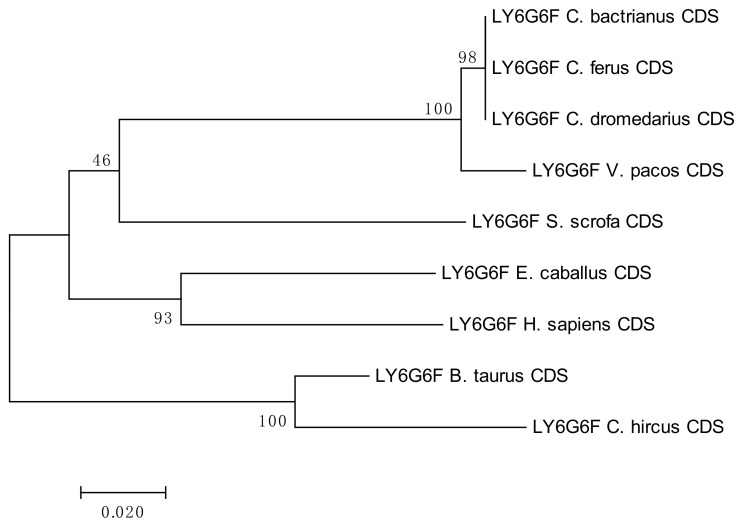
Phylogenetic tree of the *LY6G6F* CDSs from selected species. The maximum-likelihood method was used based on the Tamura 3-parameter model with gamma distribution (five categories, parameter = 0.8816). The number of bootstraps was 1000.

The results of the phylogenetic analyses summarized in Figure 3, Figure 4, Figure 5 and Figure 6 document a high level of conservation within the *LY6G6* family CDSs in the *Camelidae. LY6G6C* showed the closest evolutionary relationship with the sequence of *Sus scrofa*, while *LY6G6D* was closer to *Bos taurus* and *Capra hircus*. *LY6G6E* was close to sequences of all other included Cetartyodactyla *Sus scrofa*, *Bos taurus* and *Capra hircus,* and *LY6G6F* was also closest to *Sus scrofa*. In humans, *LY6G6E* has a status of pseudogene. Sequence similarities of CDSs used for constructing the phylogenetic trees are provided in Tables S3–S6.

### 3.3. New Annotation of the MHC Region in the Dromedary

**Figure 7:**
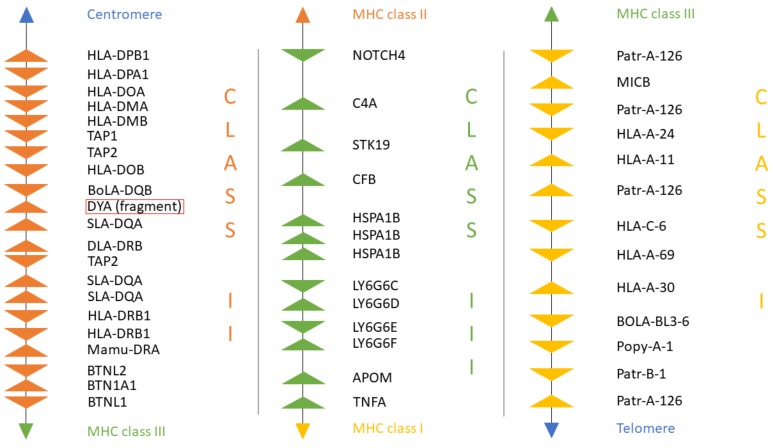
The organization of the MHC region in *C. dromedarius*. MHC class II genes are shown in orange. MHC class III genes are shown in green. MHC class I genes are shown in yellow. Spaces between genes do not represent real distances. Details of the annotation are available in Table S8.

**Figure 8:**
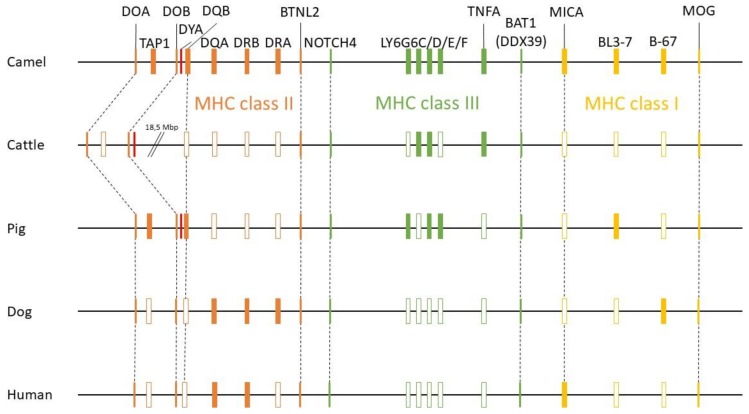
Summary of evolutionary relationships of MHC genes of dromedary, cattle, pig, dog and human. Relative gene positions are presented as rectangles. The filled rectangles highlight the closest relationships observed between the dromedary and the respective species for a given locus within the groups studied.

**Figure 9:**
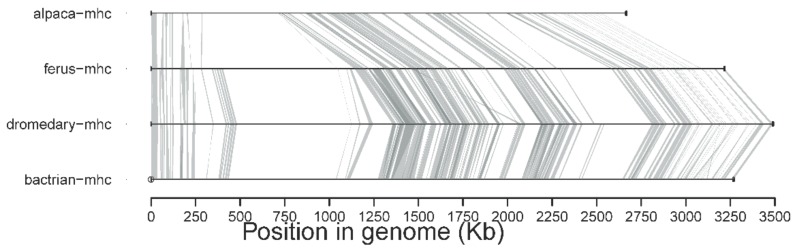
The synteny plot of the camelid MHC region showing a general structural conservation among all camelids. Assemblies of the dromedary (CamDro3) [26]; Bactrian (CamBac2, GCF_000767855.1 scaffolded with CamDro3 as a reference); wild Bactrian (CamFer2, GCF_000311805.1 scaffolded with CamDro3 as a reference); and alpaca (DNA Zoo *V. pacos*) were used.

A new version of the organization of the MHC region in the dromedary is shown in Figure 7. A complete annotation record will be available in Lado et al. [26]. A graphical summary of the evolutionary relationships for the genes studied is presented in Figure 8. The synteny plot for available camelid MHC regions is shown in Figure 9.

## 4. Discussion

This study brings new information on MHC class III sub-region genes in Old World camels. These data, along with new camel genomic resources, allowed us to construct an improved genomic map of the entire MHC region of Old World camels.

The MHC class III region, where no genes coding for antigen presenting molecules are located, represented a gap in our knowledge of the camel MHC. For bridging this gap in the map, we selected different types of class III genes with diverse immunological functions. Based on this, we could make a comparison of their variability with so far known genes from other MHC regions previously studied in the same groups of dromedary and Bactrian camels [24,25]. The data then primarily served for allowing us an overall characterization of the camelid MHC. As such, they do not represent a detailed analysis of the MHC class III region, which still is our pending task.

The *TNFA* (tumor necrosis factor alpha) is a cytokine playing a major role in the activation of both acute and chronical inflammatory processes, and contributing to the activation cascade of mother cytokines and chemokines connected with inflammation [42]. Besides this role, the *TNFA* also contributes to the regulation of apoptosis and carcinogenesis [43,44]. While there are no reports on the role of TNF-alpha and/or of the *TNFA* gene in diseases of camels, in cattle, a closely-related species, *TNFA* polymorphisms have been associated with multiple infectious diseases and with fertility [45,46,47,48]. The SNPs identified in this report thus represent potentially useful markers for analyzing various camel diseases. *TNFA,* subject to strong purifying selection [42], may be also used as a marker of a conserved part of the MHC in close physical proximity of MHC genes under positive selection pressure [48].

The LY6G6 (lymphocyte antigen 6 family, member G6) gene family is located within the MHC class III region, spanning approximately 20 kb. In humans (assembly GRCh38.p12) and mice (assembly GRCm38.p4), the family contains four individual genes, *LY6G6C, LY6G6D*, *LY6G6E*, and *LY6G6F*. LY6 proteins containing cysteine-rich domain are attached to the cell surface by a GPI anchor, which is involved in signal transduction. LY6 proteins might play a role in the hematopoietic differentiation [49].

This is a first report on the existence of this group of MHC class III genes in camels. Although little is currently known about their functions, they represent suitable anchor and marker loci for the MHC class III region. Sequences of all four selected genes showed high levels of conservation within Old World camels. SNPs were rather rare and were often observed in only one camel species; *LY6G6E* was even completely monomorphic in both species. Interestingly, while the *LY6G6E* sequence has the status of pseudogene in humans, we did not find any features of non-functional *LY6G6E* sequences in the two camel species. These findings are in agreement with those of other closely related mammals, such as *Bos taurus* and *Sus scrofa*, suggesting that this gene is probably functionally important for the Cetartiodactyla.

Having characterized selected MHC class III genes in Old World camels, we were able to build upon our previous studies of MHC class I and MHC class II regions and to update the general organization of the MHC region, describe its similarities with New World camelids, as well as with relevant mammalian species, and to point out its unique features.

The MHC class III genes studied here do not encode antigen presenting molecules and are not involved in the mechanisms of antigen presentation. For comparison, we chose *TAP1*, a gene not coding for antigen presenting molecules, but playing an important role in the process of antigen loading of the MHC class I molecules, located in the MHC class II region [50]. Certain *TAP1* variants may have a large impact on MHC class I expression and interaction with pathogens, at least in some species [51,52]. While *TNFA* and the gene family LY6 showed high levels of conservation characteristic for MHC class III loci in general, the camel *TAP1* was not only polymorphic, but the numbers of SNPs detected were higher than in the MHC class I and class II genes examined so far [24,25].

### 4.1. MHC Region Organization and Diversity in Old World Camels

The presented MHC organization is primarily based on dromedary sequences, but due to the high conservation and similarity of this region between dromedaries and Bactrian camels, we can take firm conclusions about the latter’s MHC. The overall nucleotide and amino acid sequence similarities between the two species ranged across the MHC region (delimited by the genes *TRIM27* and *RXRB*) on chromosome 20 of the new dromedary assembly CamDro3 [26], with no major differences between the three sub-regions, as seen in Figure 9. Extensive trans-species polymorphism (TSP), i.e., allele sharing, is also a typical feature of the MHC of camelids, including llamas [24,25].

The diversity of the MHC class I, class I–related and class II genes is generally lower than expected, based on a comparative analysis of multiple vertebrate species [53]. This finding is in agreement with a lower genome-wide diversity in dromedaries than in the wild and domestic Bactrian camels [28]. We were unable to make a reliable estimate of polymorphisms of class II *DQB* genes. Their exon 2 encoded amino acid sequences containing the antigen-binding site are by four amino-acids longer due to a 12 bp insertion in both camel species. They contain multiple SNPs, but we were unable to identify individual loci [24]. It is possible that certain specificities in *DQB* sequences, such as GC rich regions and/or long repeats cause technical problems as described, e.g., for the horse *DQB* region [54]. Interestingly, some of the genes encoding other than antigen presenting molecules and located in the MHC class I and II regions, such as *MICA* and *TAP1*, were found to have higher polymorphism than classical MHC class I and II genes. On the other hand, the class II *DRA* gene, monomorphic in most mammalian species, is polymorphic both in dromedaries and Bactrian camels [24]. Recently identified MHC-linked microsatellite loci also showed unusually low diversity both in dromedaries and bactrians, often with only two or three alleles [55]. Bottlenecks experienced in the evolutionary history of the three species, and also in recent times due to domestication, might be the cause of the reduced genome-wide variability in dromedaries. However, it is not clear to what extent these processes contributed to the low diversity of the immunogenome. Additionally, the reasons for higher numbers of SNPs observed in genes coding for other than antigen presenting MHC molecules remains especially puzzling.

### 4.2. Cross-Species Comparisons with New World Camelids and Other Mammals

A direct comparison with alpaca sequences presented here, as well as in our previous work [24,25], showed that the studied loci are generally highly conserved in camelids. Based on the data available so far, as seen in Figure 9, it seems that the organization of the MHC genomic region in *Vicugna pacos* is highly similar if not nearly identical to Old World camels.

When compared to other species, the physical location and the overall organization of the MHC in camels follows the common general structure of the mammalian MHC region [24]. However, phylogenetic relationships of camel MHC genes do not always follow relationships based on other (neutral) nuclear genes [24,25,38,56], suggesting that different MHC sub-regions might have followed different evolutionary pathways. In addition, so far, we have been unable to identify a *DYA* locus, specific for the *Bovidae*, the phylogenetically closest family. Although *DYA*-like sequences were previously annotated in camelid genomes, as seen in Figure 7, a more focused analysis showed that they are more similar to *DQA* rather than to *DYA* sequences, and that it was impossible to identify this region within the camel MHC physical map [38]. Like in camels, the porcine MHC class II region contains a non-functional fragment of DY gene (GenBank Gene ID: 100135048), although it is annotated as *DYB*, while the camel sequence is annotated as *DYA*. Moreover, in cattle, the physical position of the *TAP1* gene is different from other mammalian species, including camels, as seen in Figure 8. Overall, the organization of the camel MHC class II region is more similar to *Sus scrofa* rather than to *Bos taurus*.

Nucleotide sequences of genes located in the MHC class II sub-region are generally closer to dog and human, with the exception of *DQB* and *TAP1,* which are closely related to their porcine counterparts, as seen in Figure 7 [38]. On the other hand, the MHC class III genes and the LY6G6 gene family, are more related to the corresponding porcine sequences, with the exception of *LY6G6D* and *TNFA,* which are closer to the bovine sequences than to its porcine counterpart. Despite a probably common origin and close physical proximity of the LY6G6 gene family, the *LY6G6D* does not behave identically in phylogenetic trees, as seen in Figure 3, Figure 4, Figure 5 and Figure 6 [57]. It thus seems that different MHC sub-regions have not always the same evolutionary history, reflecting probably the fact that the selection pressures exerted on the immune system are different between camels and cattle.

The evolution of the MHC class I sub-region appears to be even more complex, as shown in our previous study [25]. The MHC class I locus *B-67* is closely related to the canine locus *DLA-88*, the locus *BL3-7* is related to the porcine *SLA-11*, while *MICA* is closest to the relevant human sequences. While the camel MIC genes follow the pattern of the human *MICA/B* genes, cattle have at least three functional MIC genes with unique sequences [25,58]. In contrast to these differences between camel and cattle, high similarities with the pig MHC class I genes were often observed. Especially, the MHC class I gene *BL3-7*, a locus of unclear status, highly similar to the annotated sequence *BL3-6* in alpacas, is also closely related to the locus *SLA-11* in pig, and is one MHC locus with unknown function and unusual structure [25,59]. Interestingly, we have also found close similarities between camels and pigs for two other complex immunogenomic regions, the natural killer complex (NKC) and the leukocyte receptor complex (LRC), encoding natural killer cell receptor molecules. Both regions differ from their cattle counterparts, resembling more the pig NKC and LRC, respectively [60]. However, in this context, it is important to note that the annotation of MHC class I loci from CamDro3, as presented in Figure 7, is based on calculations of their sequence similarities with other species. Taking into consideration that sequence similarities alone are often not informative for a correct assignment of their classical or non-classical status in non-model organisms, we do not have enough information for definitive conclusions about this status for class I genes in camels either.

## 5. Conclusions

This first characterization of specific MHC class III genes added to the general structure and variability of the MHC in Old World camels, allowing a synopsis of the current knowledge of the MHC region of Old World camelids. Novel phylogenetic analyses between camelids and other domestic mammals showed high conservation of this region among Old and New World camels, and a closer evolutionary relationship with pigs rather than with cattle. The knowledge of the MHCs structure, function and evolution in camels is important for understanding their immune response to diseases in their specific and extreme environments.

## Figures and Tables

**Table 1:** List of analyzed genes and numbers of individuals of *C. dromedarius* and *C. bactrianus*.

Locus	*Camelus bactrianus* (n)	*Camelus dromedarius* (n)
*TNFA*	21	22
*TAP1*	10	8
*Ly6G6C*	10	10
*Ly6G6D*	10	10
*Ly6G6E*	10	10
*Ly6G6F*	10	10

**Table 2:** List of primers used in amplification of selected major histocompatibility complex (MHC) genes in camels.

Name	Sequence 5’ → 3’	Locus	Tm (°C)	Product Length
TAP1-1-F	CATTACCCCAGTGTGGACTTCT	*TAP1*	64.4	8268
TAP1-1-R	GCAACCAAAGGAAATTGAAAAC			
LY6G6C-1-F	GGAGGGACCGTTGGAATTAT	*LY6G6C*	68.0	3491
LY6G6C-1-R	GGCGGCTTTTCTGTCAATAG			
LY6G6D-1-F	CCTCCCCTTTTATTGCCCTA	*LY6G6D*	68.0	2479
LY6G6D-1-R	CCCCATATCACTCCTTCAGC			
LY6G6E-1-F	CACAAGTGGTCACGGTCTCT	*LY6G6E*	68.0	4703
LY6G6E-1-R	GAGTGCTACTTCCCAGTCCAG			
LY6G6F-1-F	GCGTTTATCTGGGCTCTGTT	*LY6G6F*	65.0	3854
LY6G6F-1-R	CCACCCTGTCACTGGCTACT			
TNF-1-F	TGCCTGAGTGTCTGAAAGTCC	*TNFA*	66.0	3667
TNF-1-R	CCCACATACACAGCAGGAACT			

## Supplementary Materials

The supplementary materials are available online at https://www.mdpi.com/2073-4409/8/10/1200/s1.

## References

[1] Janeway C.A., Travers P., Walport M., Shlomchik M.J. (2005). Immunobiology: The Immune System in Health and Disease.

[2] Hedrick P.W., Whittam T.S., Parham P. (1991). Heterozygosity at individual amino acid sites: Extremely high levels for HLA-A and-B genes. Proc. Natl. Acad. Sci. USA.

[3] Kumánovics A., Takada T., Lindahl K.F. (2003). Genomic organization of the mammalian MHC. Annu. Rev. Immunol..

[4] Winternitz J.C., Minchey S.G., Garamszegi L.Z., Huang S., Stephens P.R., Altizer S. (2013). Sexual selection explains more functional variation in the mammalian major histocompatibility complex than parasitism. Proc. Biol. Sci..

[5] Rocha R.G., Magalhães V., López-Bao J.V., van der Loo W., Llaneza L., Alvares F., Esteves P.J., Godinho R. (2019). Alternated selection mechanisms maintain adaptive diversity in different demographic scenarios of a large carnivore. BMC Evol. Biol..

[6] Aguilar A., Roemer G., Debenham S., Binns M., Garcelon D., Wayne R.K. (2004). High MHC diversity maintained by balancing selection in an otherwise genetically monomorphic mammal. Proc. Natl. Acad. Sci. USA.

[7] Mikko S., Røed K., Schmutz S., Andersson L. (1999). Monomorphism and polymorphism at Mhc DRB loci in domestic and wild ruminants. Immunol. Rev..

[8] Doxiadis G.G., Otting N., de Groot N.G., Bontrop R.E. (2001). Differential evolutionary MHC class II strategies in humans and rhesus macaques: Relevance for biomedical studies. Immunol. Rev..

[9] Bernatchez L., Landry C. (2003). MHC studies in nonmodel vertebrates: What have we learned about natural selection in 15 years?. J. Evol. Biol..

[10] Burger P.A., Ciani E., Faye B. (2019). Old World camels in a modern world—A balancing act between conservation and genetic improvement. Anim. Genet..

[11] Wu H., Guang X., Al-Fageeh M.B., Cao J., Pan S., Zhou H., Zhang L., Abutarboush M.H., Xing Y., Xie Z. (2014). Camelid genomes reveal evolution and adaptation to desert environments. Nat. Commun..

[12] Ji R., Cui P., Ding F., Geng J., Gao H., Zhang H., Yu J., Hu S., Meng H. (2009). Monophyletic origin of domestic bactrian camel (Camelus bactrianus) and its evolutionary relationship with the extant wild camel (Camelus bactrianus ferus). Anim. Genet..

[13] Silbermayr K., Orozco-terWengel P., Charruau P., Enkhbileg D., Walzer C., Vogl C., Schwarzenberger F., Kaczensky P., Burger P.A. (2010). High mitochondrial differentiation levels between wild and domestic Bactrian camels: A basis for rapid detection of maternal hybridization. Anim. Genet..

[14] Sequencing T.B.C.G., Analysis Consortium (2012). Genome sequences of wild and domestic bactrian camels. Nat. Commun..

[15] Burger P.A. (2016). The history of Old World camelids in the light of molecular genetics. Trop. Anim. Health Pro..

[16] Wernery U., Kinne J. (2012). Foot and mouth disease and similar virus infections in camelids: A review. Rev. Sci. Tech. Oie.

[17] Dirie M.F., Abdurahman O. (2003). Observations on little known diseases of camels (Camelus dromedarius) in the Horn of Africa. Rev. Sci. Tech. Oie.

[18] Al Kanhal H.A. (2010). Compositional, technological and nutritional aspects of dromedary camel milk. Int. Dairy J..

[19] Ali A., Baby B., Vijayan R. (2019). Camel Genome-from Desert to Medicine. Front. Genet..

[20] Muyldermans S. (2001). Single domain camel antibodies: Current status. Rev. Mol. Biotech..

[21] Ciccarese S.M., Burger P., Ciani E., Castelli V., Linguiti G., Plasil M., Massari S., Horin P., Antonacci R. (2019). The camel adaptive immune receptors repertoire as a singular example of structural and functional genomics. Front. Genet..

[22] Antczak D. (2013). Major histocompatibility complex genes of the dromedary camel. Proceedings of the Qatar Foundation Annual Research Conference, Doha, Qatar, 24–25, November, 2013.

[23] Avila F., Baily M.P., Perelman P., Das P.J., Pontius J., Chowdhary R., Owens E., Johnson W.E., Merriwether D.A., Raudsepp T. (2014). A comprehensive whole-genome integrated cytogenetic map for the alpaca (Lama pacos). Cytogenet. Genome Res..

[24] Plasil M., Mohandesan E., Fitak R.R., Musilova P., Kubickova S., Burger P.A., Horin P. (2016). The major histocompatibility complex in Old World camelids and low polymorphism of its class II genes. BMC Genomics.

[25] Plasil M., Wijkmark S., Elbers J.P., Oppelt J., Burger P., Horin P. (2019). The major histocompatibility complex of Old World camelids: Class I and class I-related genes. HLA.

[26] Lado S., Elbers J.P., Rogers M.F., Perelman P.L., Proskuryakova A.A., Serdyukova N.A., Johnson W.E., Horin P., Corander J., Murphy D. Reference-guided assembly of two Old World camel genomes and genomic diversity of Old World camelid immune response genes.

[27] Elbers J.P., Rogers M.F., Perelman P.L., Proskuryakova A.A., Serdyukova N.A., Johnson W.E., Horin P., Corander J., Murphy D., Burger P.A. (2019). Improving Illumina assemblies with Hi-C and long reads: An example with the North African dromedary. Mol. Ecol. Resour..

[28] Fitak R.R., Mohandesan E., Corander J., Burger P.A. (2016). The de novo genome assembly and annotation of a female domestic dromedary of North African origin. Mol. Ecol. Resour..

[29] Putnam N.H., O’Connell B.L., Stites J.C., Rice B.J., Blanchette M., Calef R., Troll C.J., Fields A., Hartley P.D., Sugnet C.W. (2016). Chromosome-scale shotgun assembly using an in vitro method for long-range linkage. Genome Res..

[30] English A.C., Richards S., Han Y., Wang M., Vee V., Qu J., Qin X., Muzny D.M., Reid J.G., Worley K.C. (2012). Mind the gap: Upgrading genomes with Pacific Biosciences RS long-read sequencing technology. PLoS ONE.

[31] Walker B.J., Abeel T., Shea T., Priest M., Abouelliel A., Sakthikumar S., Cuomo C.A., Zeng Q., Wortman J., Young S.K. (2014). Pilon: An integrated tool for comprehensive microbial variant detection and genome assembly improvement. PLoS ONE.

[32] Tarasov A., Vilella A.J., Cuppen E., Nijman I.J., Prins P. (2015). Sambamba: Fast processing of NGS alignment formats. Bioinformatics.

[33] Jackman S.D., Vandervalk B.P., Mohamadi H., Chu J., Yeo S., Hammond S.A., Jahesh G., Khan H., Coombe L., Warren R.L. (2017). ABySS 2.0: Resource-efficient assembly of large genomes using a Bloom filter. Genome Res..

[34] Kumar S., Stecher G., Tamura K. (2016). MEGA7: Molecular evolutionary genetics analysis version 7.0 for bigger datasets. Mol. Biol. Evol..

[35] Tamura K. (1992). Estimation of the number of nucleotide substitutions when there are strong transition-transversion and G+ C-content biases. Mol. Biol. Evol..

[36] Jukes T.H., Cantor C.R. (1969). Evolution of protein molecules. Mammal. Prot. Metab..

[37] Kimura M. (1980). A simple method for estimating evolutionary rates of base substitutions through comparative studies of nucleotide sequences. J Mol. Evol..

[38] Plasil M. (2018). Comparative genomics of the major histocompatibility complex MHC. Ph.D. Thesis.

[39] Cantarel B.L., Korf I., Robb S.M., Parra G., Ross E., Moore B., Holt C., Alvarado A.S., Yandell M. (2008). MAKER: An easy-to-use annotation pipeline designed for emerging model organism genomes. Genome Res..

[40] Holt C., Yandell M. (2011). MAKER2: An annotation pipeline and genome-database management tool for second-generation genome projects. BMC Bioinformatics.

[41] Farrer R.A. (2017). Synima: A Synteny imaging tool for annotated genome assemblies. BMC Bioinform..

[42] Chu W.M. (2013). Tumor necrosis factor. Cancer Lett..

[43] Pan S., An P., Zhang R., He X., Yin G., Min W. (2002). Etk/Bmx as a tumor necrosis factor receptor type 2-specific kinase: Role in endothelial cell migration and angiogenesis. Mol. Cell. Biol..

[44] Odbileg R., Konnai S., Ohashi K., Onuma M. (2005). Molecular cloning and phylogenetic analysis of inflammatory cytokines of Camelidae (llama and camel). J. Vet. Med. Sci..

[45] Ranjan S., Bhushan B., Panigrahi M., Kumar A., Deb R., Kumar P., Sharma D. (2015). Association and expression analysis of single nucleotide polymorphisms of partial tumor necrosis factor alpha gene with mastitis in crossbred cattle. Anim. Biotechnol..

[46] Lendez P.A., Passucci J.A., Poli M.A., Gutierrez S.E., Dolcini G.L., Ceriani M.C. (2015). Association of TNF-α gene promoter region polymorphisms in bovine leukemia virus (BLV)-infected cattle with different proviral loads. Arch. Virol..

[47] Kawasaki Y., Aoki Y., Magata F., Miyamoto A., Kawashima C., Hojo T., Okuda K., Shirasuna K., Shimizu T. (2014). The effect of single nucleotide polymorphisms in the tumor necrosis factor-α gene on reproductive performance and immune function in dairy cattle. J. Reprod. Develop..

[48] Seitzer U., Gerdes J., Müller-Quernheim J. (2001). Genotyping in the MHC locus: Potential for defining predictive markers in sarcoidosis. Resp. Res..

[49] Mallya M., Campbell R.D., Aguado B. (2006). Characterization of the five novel Ly-6 superfamily members encoded in the MHC, and detection of cells expressing their potential ligands. Protein. Sci..

[50] Trowsdale J., Hanson I., Mockridge I., Beck S., Townsendt A., Kelly A. (1990). Sequences encoded in the class II region of the MHC related to the ’ABC’ superfamily of transporters. Nature.

[51] Kaufman J. (2015). Co-evolution with chicken class I genes. Immunol. Rev..

[52] Praest P., Luteijn R.D., Brak-Boer I.G.J., Lanfermeijer J., Hoelen H., Ijgosse L., Costa A.I., Gorham R.D., Lebbink R.J., Wiertz E. (2018). The influence of TAP1 and TAP2 gene polymorphisms on TAP function and its inhibition by viral immune evasion proteins. Mol. Immunol..

[53] Kulski J.K., Shiina T., Anzai T., Kohara S., Inoko H. (2002). Comparative genomic analysis of the MHC: The evolution of class I duplication blocks, diversity and complexity from shark to man. Immunol. Rev..

[54] Viļuma A., Mikko S., Hahn D., Skow L., Andersson G., Bergström T.F. (2017). Genomic structure of the horse major histocompatibility complex class II region resolved using PacBio long-read sequencing technology. Sci. Rep..

[55] Wijacki J. (2019). Personal communication.

[56] Wang Q., Yang C. (2013). The phylogeny of the Cetartiodactyla based on complete mitochondrial genomes. Int. J. Biol..

[57] Mallya M., Campbell R.D., Aguado B. (2002). Transcriptional analysis of a novel cluster of LY-6 family members in the human and mouse major histocompatibility complex: Five genes with many splice forms. Genomics.

[58] Birch J., Sanjuan C.D.J., Guzman E., Ellis S.A. (2008). Genomic location and characterisation of MIC genes in cattle. Immunogenetics.

[59] Renard C., Vaiman M., Chiannilkulchai N., Cattolico L., Robert C., Chardon P. (2001). Sequence of the pig major histocompatibility region containing the classical class I genes. Immunogenetics.

[60] Futas J., Oppelt J., Jelinek A., Elbers J.P., Wijacki J., Knoll A., Burger P.A., Horin P. (2019). Natural killer cell receptor genes in camels: Another mammalian model. Front. Genet..

