# Supplementary material for: The Major Histocompatibility Complex of Old World Camels—A Synopsis
Source: Cells. 2019 Oct 5;8(10):1200. doi: 10.3390/cells8101200 (PMC6829570; doi:10.3390/cells8101200)
Supplement: Supplementary file 1 [file cells-08-01200-s001.zip › Table S5.docx]

Table S5: Sequences used for the construction of *LY6G6E* phylogenetic tree. Nucleotide and polypeptide identity is compared to the *LY6G6E* CDS of *C. bactrianus* (XM_010961503.1:74-475).

| Locus | ID | Nucleotide identity [%] | Polypeptide identity [%] |
| --- | --- | --- | --- |
| *LY6G6E* CDS *Camelus dromedarius* | XM_010978129.1:74-475 | 100 | 100 |
| *LY6G6E* CDS *Camelus ferus* | XM_014554238.1:63-464 | 100 | 100 |
| *LY6G6E* CDS *Vicugna pacos* | XM_006215414.2:54-455 | 99.5 | 99.3 |
| *LY6G6E* CDS *Bos taurus* | NM_001081520.2:121-597 | 72.1 | 66.7 |
| *LY6G6E* CDS *Sus scrofa* | XM_021098454.1:395-796 | 88.6 | 83.6 |
| *LY6G6E* CDS *Capra hircus* | XM_018038619.1:252-728 | 72.5 | 68.6 |
| *LY6G6E* CDS *Homo sapiens* | NR_003673.3 | 50.2 | 41.7 |
