# Supplementary material for: The Major Histocompatibility Complex of Old World Camels—A Synopsis
Source: Cells. 2019 Oct 5;8(10):1200. doi: 10.3390/cells8101200 (PMC6829570; doi:10.3390/cells8101200)
Supplement: Supplementary file 1 [file cells-08-01200-s001.zip › Table S6.docx]

Table S6: Sequences used for the construction of *LY6G6F* phylogenetic tree. Nucleotide and polypeptide identity is compared to the *LY6G6F* CDS of *C. bactrianus* (XM_010961500.1:20-919).

| Locus | ID | Nucleotide identity [%] | Polypeptide identity [%] |
| --- | --- | --- | --- |
| *LY6G6F* CDS *Camelus dromedarius* | XM_010978125.1:20-919 | 99.8 | 99.7 |
| *LY6G6F* CDS *Camelus ferus* | XM_006178771.2:20-919 | 99.9 | 100 |
| *LY6G6F* CDS *Vicugna pacos* | XM_006215334.2:20-919 | 98 | 97 |
| *LY6G6F* CDS *Bos taurus* | NM_001076194.1:28-927 | 86.3 | 79.3 |
| *LY6G6F* CDS *Capra hircus* | XM_005696664.2 | 72.3 | 63.9 |
| *LY6G6F* CDS *Equus caballus* | XM_005603740.3:672-1820 | 68.2 | 62.9 |
| *LY6G6F* CDS *Sus scrofa* | NM_001195347.1:25-924 | 86.8 | 80 |
| *LY6G6F* CDS *Homo sapiens* | NM_001003693.2:45-938 | 83.6 | 78.7 |
