# Supplementary material for: The Major Histocompatibility Complex of Old World Camels—A Synopsis
Source: Cells. 2019 Oct 5;8(10):1200. doi: 10.3390/cells8101200 (PMC6829570; doi:10.3390/cells8101200)
Supplement: Supplementary file 1 [file cells-08-01200-s001.zip › Table S7.docx]

Table S7: The frequency of heterozygotes and translational status in detected SNPs from *TAP1* CDS in *C. bactrianus* and *C. dromedarius*.

| SNP position | *Camelus bactrianus* (n=10) | *Camelus dromedarius* (n=8) | Synonymous (S) x Non-synonymous (N) substitution |
| --- | --- | --- | --- |
| 210 | - | 0.125 | S |
| 541 | 0.5 | - | N |
| 544 | 0.8 | 0.25 | N |
| 790 | 0.1 | - | N |
| 871 | 0.7 | 0.375 | N |
| 894 | - | 0.125 | N |
| 969 | 0.9 | - | S |
| 994 | - | 0.125 | N |
| 1099 | 0.1 | - | S |
| 1134 | - | 0.125 | S |
| 1219 | 0.8 | 0.375 | N |
| 1451 | 0.1 | - | N |
| 1491 | 0.8 | 0.375 | S |
| 1568 | 0.8 | 0.375 | N |
| 1602 | 0.5 | - | S |
| 1931 | - | 0.25 | N |
| 1974 | 0.8 | 0.375 | S |
| 2107 | 0.1 | 0.25 | N |
| 2234 | 0.4 | - | N |
