# Supplementary material for: The Major Histocompatibility Complex of Old World Camels—A Synopsis
Source: Cells. 2019 Oct 5;8(10):1200. doi: 10.3390/cells8101200 (PMC6829570; doi:10.3390/cells8101200)
Supplement: Supplementary file 1 [file cells-08-01200-s001.zip › Table S8.docx]

Table S8: List of annotated genes with bp positions used to construct schematic overview of the camelid MHC region in Figure 7.

| Locus | Start position | End position | CamDro3 ID |
| --- | --- | --- | --- |
| HLA-DPB1 | 20675921 | 20682100 | Cadr_00022016 |
| HLA-DPA1 | 20683901 | 20692996 | Cadr_00022017 |
| HLA-DOA | 20697255 | 20702100 | Cadr_00022018 |
| HLA-DMA | 20735209 | 20738671 | Cadr_00022020 |
| HLA-DMB | 20745803 | 20751818 | Cadr_00022021 |
| TAP1 | 20791956 | 20803529 | Cadr_00022023 |
| TAP2 | 20809569 | 20819965 | Cadr_00022025 |
| HLA-DOB | 20826010 | 20832443 | Cadr_00022026 |
| BoLA-DQB | 20836350 | 20839482 | Cadr_00022027 |
| DYA (fragment)* | 20853011 | 20853262 | Not part of current annotation record |
| SLA-DQA | 20852606 | 20853262 | Cadr_00022028 |
| DLA-DRB | 20859806 | 20873351 | Cadr_00022030 |
| TAP2 | 20874217 | 20874498 | Cadr_00022031 |
| SLA-DQA | 20919544 | 20925173 | Cadr_00022033 |
| SLA-DQA | 20972730 | 20977091 | Cadr_00022035 |
| HLA-DRB1 | 21028045 | 21035692 | Cadr_00022036 |
| HLA-DRB1 | 21044738 | 21051340 | Cadr_00022037 |
| Mamu-DRA | 21058242 | 21063499 | Cadr_00022038 |
| BTNL2 | 21071227 | 21081361 | Cadr_00022039 |
| BTN1A1 | 21084001 | 21093991 | Cadr_00022040 |
| BTNL1 | 21098283 | 21107744 | Cadr_00022041 |
| NOTCH4 | 21981267 | 22001999 | Cadr_00022054 |
| C4A | 22121043 | 22134861 | Cadr_00022065 |
| STK19 | 22135545 | 22143157 | Cadr_00022067 |
| CFB | 22157682 | 22179036 | Cadr_00022072 |
| HSPA1B | 22247651 | 22255041 | Cadr_00022077 |
| HSPA1B | 22265027 | 22265506 | Cadr_00022078 |
| HSPA1B | 22265865 | 22266572 | Cadr_00022079 |
| LY6G6C | 22333284 | 22336530 | Cadr_00022085 |
| LY6G6D* | 22337234 | 22338912 | Not part of current annotation record |
| LY6G6E | 22338720 | 22341713 | Cadr_00022086 |
| LY6G6F | 22343399 | 22351773 | Cadr_00022087 |
| APOM | 22379505 | 22394977 | Cadr_00022092 |
| TNFA | 22446240 | 22448489 | Cadr_00022101 |
| Patr-A-126 | 22493300 | 22505815 | Cadr_00022105 |
| MICB | 22507836 | 22522727 | Cadr_00022106 |
| Patr-A-126 | 23037821 | 23047365 | Cadr_00022139 |
| HLA-A-24 | 23100051 | 23104137 | Cadr_00022140 |
| HLA-A-11 | 23134418 | 23136926 | Cadr_00022145 |
| Patr-A-126 | 23166399 | 23170132 | Cadr_00022147 |
| HLA-C-6 | 23246587 | 23248363 | Cadr_00022148 |
| HLA-A-69 | 23254707 | 23263729 | Cadr_00022149 |
| HLA-A-30 | 23262020 | 23272548 | Cadr_00022150 |
| BOLA-BL3-6 | 23290925 | 23292196 | Cadr_00022152 |
| Popy-A-1 | 23335207 | 23336312 | Cadr_00022155 |
| Patr-B-1 | 23350555 | 23351764 | Cadr_00022156 |
| Patr-A-126 | 23401819 | 23408382 | Cadr_00022160 |

*Loci not present in current annotation record of CamDro3 assembly were manually BLASTed and their respective positions are presented.
