# Supplementary material for: The Major Histocompatibility Complex of Old World Camels—A Synopsis
Source: Cells. 2019 Oct 5;8(10):1200. doi: 10.3390/cells8101200 (PMC6829570; doi:10.3390/cells8101200)
Supplement: Supplementary file 1 [file cells-08-01200-s001.zip › Table S1.docx]

Table S1: Sequences used for the construction of *TAP1* phylogenetic tree. Nucleotide and polypeptide identity is compared to the *TAP1* CDS of *C. dromedarius* (XM_010994582.1:25-2265).

| Locus | ID | Nucleotide identity [%] | Polypeptide identity [%] |
| --- | --- | --- | --- |
| *TAP1* CDS *Camelus bactrianus* | XM_010949116.1:91-2325 | 98.7 | 98 |
| *TAP1* CDS *Camelus ferus* | XM_014555673.1:79-2295 | 96.7 | 96.5 |
| *TAP1* CDS *Vicugna pacos* | XM_006202144.2:93-2333 | 98.4 | 97.5 |
| *TAP1* CDS *Bos taurus* | NM_001098058.1 | 84.2 | 78.2 |
| *TAP1* CDS *Capra hircus* | XM_005696445.3:302-2554 | 83.3 | 78.2 |
| *TAP1* CDS *Equus caballus* | XM_001496035.4:395-2647 | 87.2 | 83.6 |
| *TAP1* CDS *Sus scrofa* | XM_021098370.1:222-2435 | 85.8 | 82.5 |
| *TAP1* CDS *Homo sapiens* | NM_000593.5:156-2582 | 77.6 | 73.2 |
