# Supplementary material for: The Major Histocompatibility Complex of Old World Camels—A Synopsis
Source: Cells. 2019 Oct 5;8(10):1200. doi: 10.3390/cells8101200 (PMC6829570; doi:10.3390/cells8101200)
Supplement: Supplementary file 1 [file cells-08-01200-s001.zip › Table S2.docx]

Table S2: Sequences used for the construction of *TNFA* phylogenetic tree. Nucleotide and polypeptide identity is compared to the *TNFA* CDS of *C. bactrianus* (XM_010961535.1:191-892).

| Locus | ID | Nucleotide identity [%] | Polypeptide identity [%] |
| --- | --- | --- | --- |
| *TNFA* CDS *Camelus dromedarius* | XM_010978144.1:191-892 | 99.6 | 99.2 |
| *TNFA* CDS *Camelus ferus* | XM_006178751.2:191-892 | 99.6 | 99.2 |
| *TNFA* CDS *Vicugna pacos* | XM_006215316.1:191-892 | 99 | 99.6 |
| *TNFA* CDS *Bos taurus* | NM_173966.3:194-898 | 84.3 | 79.2 |
| *TNFA* CDS *Ovis aries* | NM_001024860.1:173-877 | 84.3 | 80 |
| *TNFA* CDS *Equus caballus* | NM_001081819.2:36-740 | 86.5 | 83.1 |
| *TNFA* CDS *Sus scrofa* | NM_214022.1:184-882 | 86.3 | 85.2 |
| *TNFA* CDS *Canis lupus familiaris* | NM_001003244.4:1-702 | 85.5 | 83.1 |
| *TNFA* CDS *Homo sapiens* | NM_000594.3:176-877 | 85.5 | 82.2 |
| *TNFA* CDS *Mus musculus* | NM_013693.3:168-875 | 76.8 | 72.6 |
