# Supplementary material for: The Major Histocompatibility Complex of Old World Camels—A Synopsis
Source: Cells. 2019 Oct 5;8(10):1200. doi: 10.3390/cells8101200 (PMC6829570; doi:10.3390/cells8101200)
Supplement: Supplementary file 1 [file cells-08-01200-s001.zip › Table S3.docx]

Table S3: Sequences used for the construction of *LY6G6C* phylogenetic tree. Nucleotide and polypeptide identity is compared to the *LY6G6C* CDS of *C. bactrianus* ((XM_010961499.1:52-429).

| Locus | ID | Nucleotide identity [%] | Polypeptide identity [%] |
| --- | --- | --- | --- |
| *LY6G6C* CDS *Camelus dromedarius* | XM_010978124.1:52-429 | 99.7 | 99.2 |
| *LY6G6C* CDS *Camelus ferus* | XM_006178772.2:56-433 | 99.7 | 99.2 |
| *LY6G6C* CDS *Vicugna pacos* | XM_006215335.2:49-426 | 98.9 | 98.4 |
| *LY6G6C* CDS *Bos taurus* | BC126760.1:55-432 | 86.5 | 81 |
| *LY6G6C* CDS *Sus scrofa* | XM_021098457.1:907-1284 | 91.2 | 93.7 |
| *LY6G6C* CDS *Equus caballus* | XM_023624628.1:692-1066 | 94.4 | 93.7 |
| *LY6G6C* CDS *Capra hircus* | XM_005696575.3:84-461 | 86 | 80.2 |
| *LY6G6C* CDS *Homo sapiens* | NM_025261.2:56-433 | 91.3 | 87.3 |
