# Supplementary material for: The Major Histocompatibility Complex of Old World Camels—A Synopsis
Source: Cells. 2019 Oct 5;8(10):1200. doi: 10.3390/cells8101200 (PMC6829570; doi:10.3390/cells8101200)
Supplement: Supplementary file 1 [file cells-08-01200-s001.zip › Table S4.docx]

Table S4: Sequences used for the construction of *LY6G6D* phylogenetic tree. Nucleotide and polypeptide identity is compared to the *LY6G6D* CDS of *C. bactrianus* (XM_010961504.1:32-457).

| Locus | ID | Nucleotide identity [%] | Polypeptide identity [%] |
| --- | --- | --- | --- |
| *LY6G6D* CDS *Camelus dromedarius* | XM_010978130.1:32-457 | 100 | 100 |
| *LY6G6D* CDS *Camelus ferus* | XM_006178770.2:39-464 | 100 | 100 |
| *LY6G6D* CDS *Vicugna pacos* | XM_006215333.2:59-484 | 98.6 | 99.3 |
| *LY6G6D* CDS *Bos taurus* | XM_003587697.4:166-591 | 87.1 | 87.3 |
| *LY6G6D* CDS *Equus caballus* | XM_005603741.3:65-478 | 85.5 | 81.1 |
| *LY6G6D* CDS *Capra hircus* | XM_005696576.2:40-465 | 88.7 | 88 |
| *LY6G6D* CDS *Sus scrofa* | NM_001195349.2:47-472 | 85.5 | 80.4 |
| *LY6G6D* CDS *Homo sapiens* | NM_021246.2 | 78.2 | 71.1 |
